# Multiple convergent events created a nominal widespread species: *Triplophysa stoliczkae* (Steindachner, 1866) (Cobitoidea: Nemacheilidae)

**DOI:** 10.1186/s12862-019-1503-3

**Published:** 2019-09-04

**Authors:** Chenguang Feng, Yongtao Tang, Sijia Liu, Fei Tian, Cunfang Zhang, Kai Zhao

**Affiliations:** 1 Key Laboratory of Adaptation and Evolution of Plateau Biota, and Laboratory of Plateau Fish Evolutionary and Functional Genomics, and Qinghai Key Laboratory of Animal Ecological Genomics, Northwest Institute of Plateau Biology, Chinese Academy of Sciences, No. 23 Xinning Road, Xining, 810008 Qinghai China; 2 Center for Ecological and Environmental Sciences, Key Laboratory for Space Bioscience and Biotechnology, Northwestern Polytechnical University, Xi’an, 710072 Shaanxi China; 3 University of Chinese Academy of Sciences, Beijing, 100049 China

**Keywords:** Convergent evolution, Phylogeny, Qinghai-Tibetan plateau, Systematics, Tibetan loach

## Abstract

**Background:**

*Triplophysa stoliczkae* is the most widespread species in the genus *Triplophysa* and may have originated from morphological convergence. To understand the evolutionary history of *T. stoliczkae*, we employed a multilocus approach to investigate the phylogenetics and the morphological evolution of *T. stoliczkae* on the Qinghai-Tibetan Plateau.

**Results:**

All phylogenetic analyses (two mitochondrial and five nuclear loci), a genealogical sorting index and species tree inferences suggested that *T. stoliczkae* consists of distinct lineages that were not closest relatives. The time estimation indicated that the divergence events between “*T. stoliczkae*” and other *Triplophysa* species occurred from approximately 0.10 to 4.51 Ma. The ancestral state analyses supported the independent evolution of *T. stoliczkae* morphology in distinct lineages. The morphometric analysis and convergence estimates demonstrated significant phenotypic convergence among “*T. stoliczkae*” lineages.

**Conclusions:**

*Triplophysa stoliczkae* includes 4 different lineages with similar morphologies. The increasingly harsh environments that have occurred since the Pliocene have driven the occurrences of scrape-feeding fish in the genus *Triplophysa*. Morphological adaptations associated with scrape-feeding behavior resulted in convergences and the artificial lumping of four different species in the nominal taxon *T. stoliczkae*. A taxonomic revision for *T. stoliczkae* is needed.

**Electronic supplementary material:**

The online version of this article (10.1186/s12862-019-1503-3) contains supplementary material, which is available to authorized users.

## Background

Convergent evolution, the independent origination of analogous biological traits, is pervasive in the evolution of life and has long been regarded as evidence of adaptation [1–5]. Generally, convergent evolution is common in species-rich communities, especially when the number of species exceeds the available niches [6–8]. Furthermore, convergent evolution creates similar phenotypes that resulted in errors in the taxonomy [5, 9, 10]. Understanding convergent evolution may help us better understand the evolutionary history and resolve the taxonomy dilemma.

The genus *Triplophysa* (Cobitoidea: Nemacheilidae), a species-rich and taxonomically unstable group, is an important component of the ichthyofauna on the Qinghai-Tibetan Plateau (QTP) [11, 12]. *Triplophysa stoliczkae* (Steindachner, 1866) is the most widely distributed species in this genus. It lives mainly in the streams of the QTP and its peripheral regions. As a scrape-feeding fish, *T. stoliczkae* has the typical characteristics of a broadened sharp lower jaw, screw-like intestine and degenerated posterior chamber of its air bladder (PCAB) (Additional file 1: Figures S1 and S2). Due to morphological variations among populations, *T. stoliczkae* has long been a controversial subject in taxonomy [11–13]. Nevertheless, *T. stoliczkae* is currently considered to be a single, widespread species with a geographic range that covers the entire QTP. The evolutionary history of QTP ichthyofauna has been shaped by the history of river drainages [14]. Generally, the QTP fish with a late phylogenetic position have had a limited distribution area because of geographical barriers. However, the mitochondrial data have suggested that *T. stoliczkae* is not an ancient lineage [14–16]. Moreover, the current habitats of *T. stoliczkae* have been disconnected from each other by ancient and persistent orogeny [17]. The conflict between the late occurrence and wide distribution of *T. stoliczkae* could be explained by two possible scenarios. First, convergent evolution creates similar morphologies that resulted in errors in the definition of *T. stoliczkae*. Second, it is possible, although unlikely, that *T. stoliczkae* evolved once and then spread to different river systems.

**Fig. 1:**
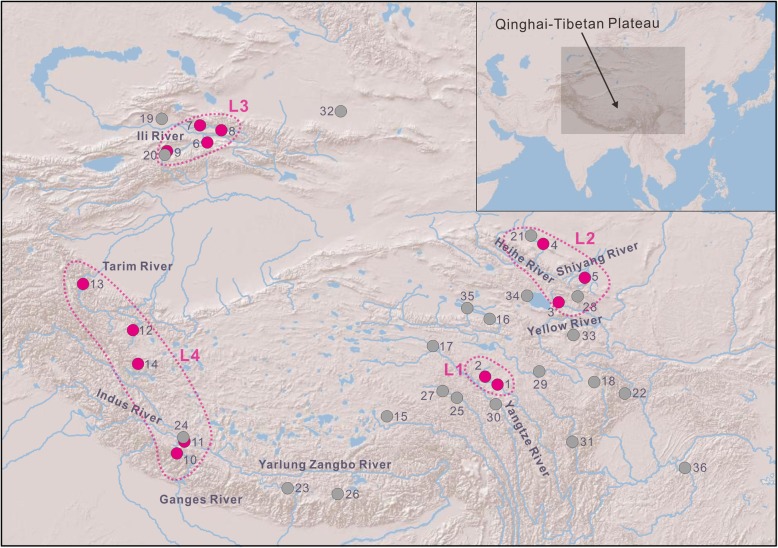
Sampling sites and the water systems. Rose-red circles denote the localities of *Triplophysa stoliczkae*, and gray circles represent the localities of the other *Triplophysa* species. Site numbers are detailed in Table 1. Light blue patches and lines depict the water systems. Rose-red dashed lines (L1 to L4) correspond to *T. stoliczkae*-1 to *T. stoliczkae*-4 in Figs. 2-5

To determine the evolutionary history of *T. stoliczkae*, we employ a multilocus approach to analyze individuals from almost all of the distribution ranges. We also use many other *Triplophysa* species from various water systems (Fig. 1) to ensure robust results. By inferring the phylogenetic relationships, quantifying genealogical sorting, and dating the divergence, we attempt to answer two questions: (1) whether *T. stoliczkae* is monophyletic and (2) when and how many times divergence events occurred in *T. stoliczkae*. Moreover, under a nonmonophyly scenario, an ancestral state analysis indicates that the occurrence of *T. stoliczkae* morphology is based on (a) multiple independent innovations or (b) the remains of ancient traits. A morphometric analysis and convergence estimate further quantify the phenotypic convergence of “*T. stoliczkae*”. Finally, together with the geologic climate background, we try to understand the evolution of *T. stoliczkae*.

## Methods

### Samples and laboratory procedures

With the permission of the fisheries departments of the Chinese government, we carried out the sampling to collect protected *Triplophysa*. All animal experiments were approved by the Ethics Committee of the Northwest Institute of Plateau Biology, Chinese Academy of Sciences. A total of 49 specimens of *Triplophysa* containing 26 specimens of *T. stoliczkae* and 23 other *Triplophysa* species from 36 sites were analyzed (Fig. 1; Table 1). Based on a previous study [16], we selected *Triplophysa siluroides* and *Triplophysa robusta* as primary the outgroup taxa and used *Triplophysa rosa* as the secondary outgroup species. Specimens of *T. stoliczkae* were collected from 8 water systems on and near the QTP: Yangtze River, Yellow River, Shiyang River, Heihe River, Ili River, Ganges River, Indus River, and Tarim River. All collected specimens were euthanized after identification. They were placed in a dry ice box for rapid hypothermic anesthesia within about 20 s. Then, they were preserved in 95% ethanol for laboratory works. Voucher specimens were archived in the collection of the Northwest Institute of Plateau Biology (NWIPB), Chinese Academy of Sciences.

**Table 1:** Samples of *Triplophysa stoliczkae* and other *Triplophysa* species used in this study. The locality IDs correspond to those in Fig. 1. The group IDs correspond to the labels of *T. stoliczkae* in Fig. 2

Sample ID	Species	Voucher ID	Group ID	Locality ID	Locality	Water systems
Focal taxon						
1	*Triplophysa stoliczkae*	NWIPB1307007	1	1	Zhaqu River, Chindu County	Yangtze River
2	*T*. *stoliczkae*	NWIPB1307052	1	2	Zhaqu River, Chindu County	Yangtze River
3	*T*. *stoliczkae*	NWIPB1108034	1	1	Zhaqu River, Chindu County	Yangtze River
4	*T*. *stoliczkae*	NWIPB1307005	1	2	Zhaqu River, Chindu County	Yangtze River
5	*T*. *stoliczkae*	NWIPB1307016	1	2	Zhaqu River, Chindu County	Yangtze River
6	*T*. *stoliczkae*	NWIPB1208003	2	3	Huangshui River, Haiyan County	Yellow River
7	*T*. *stoliczkae*	NWIPB1208004	2	3	Huangshui River, Haiyan County	Yellow River
8	*T*. *stoliczkae*	NWIPB1205081	2	4	Heihe River, Zhangye City	Heihe River
9	*T*. *stoliczkae*	NWIPB1205082	2	4	Heihe River, Zhangye City	Heihe River
10	*T*. *stoliczkae*	NWIPB1205019	2	5	Zamu River, Wuwei City	Shiyang River
11	*T*. *stoliczkae*	NWIPB1205020	2	5	Zamu River, Wuwei City	Shiyang River
12	*T*. *stoliczkae*	NWIPB1305148	3	6	Kunes River, Xinyuan County	Ili River
13	*T*. *stoliczkae*	NWIPB1305065	3	7	Kashi River, Nilka County	Ili River
14	*T*. *stoliczkae*	NWIPB1305043	3	8	Kashi River, Nilka County	Ili River
15	*T*. *stoliczkae*	NWIPB1305045	3	8	Kashi River, Nilka County	Ili River
16	*T*. *stoliczkae*	NWIPB1305111	3	6	Kunes River, Xinyuan County	Ili River
17	*T*. *stoliczkae*	NWIPB1305125	3	9	Tekes River, Tekes County	Ili River
18	*T*. *stoliczkae*	NWIPB1305127	3	9	Tekes River, Tekes County	Ili River
19	*T*. *stoliczkae*	NWIPB1106007	4	10	Kongque River, Purang County	Ganges River
20	*T*. *stoliczkae*	NWIPB1106008	4	10	Kongque River, Purang County	Ganges River
21	*T*. *stoliczkae*	NWIPB1106001	4	11	Lake Manasarovar, Purang County	Indus River
22	*T*. *stoliczkae*	NWIPB1106002	4	11	Lake Manasarovar, Purang County	Indus River
23	*T*. *stoliczkae*	NWIPB1007084	4	12	Qaraqash River, Pishan County	Tarim River
24	*T*. *stoliczkae*	NWIPB1007083	4	13	Yarkand River, Yecheng County	Tarim River
25	*T*. *stoliczkae*	NWIPB1407013	4	14	Changchuan River, Rutog County	Indus River
26	*T*. *stoliczkae*	NWIPB1407014	4	14	Changchuan River, Rutog County	Indus River
Other *Triplophysa* species						
27	*T. rotundiventris*	NWIPB1107006		15	Nagqu River, Amdo Zong	Nujiang River
28	*T. chondrostoma*	NWIPB0707033		16	Xiangride River, Dulan County	Qaidam River
29	*T. leptosoma*	NWIPB1307140		17	Sewu River, Qumalai County	Yangtze River
30	*T. orientalis*	NWIPB1505578		18	Heihe River, Ruoergai County	Yellow River
31	*T. strauchii*	NWIPB1305216		19	Sandao River, Qorghas County	Ili River
32	*T. dorsalis*	NWIPB1305232		20	Tekes River, Tekes County	Ili River
33	*T. tenuis*	NWIPB1250170		21	Heihe River, Zhangye City	Heihe River
34	*T. bleekeri*	NWIPB1505622		22	Danpu River, Wenxian County	Yangtze River
35	*T. brevicauda*	NWIPB1106029		23	Pengqu River, Nyalam County	Ganges River
36	*T. tibetana*	NWIPB1106067		24	Lake Manasarovar, Purang County	Indus River
37	*T. aliensis*	NWIPB1106031		24	Lake Manasarovar, Purang County	Indus River
38	*T. stenura*	NWIPB1108064		25	Zhaqu River, Nangqian County	Lancang River
39	*T. stewarti*	NWIPB1107007		26	Lake Duoqing, Kangmar County	Lake Duoqing
40	*T. microps*	NWIPB1307038		27	Zhaqu River, Zadoi County	Lancang River
41	*T. robusta*	NWIPB1205817		28	Datong River, Menyuan County	Yellow River
42	*T. siluroides*	NWIPB1250382		29	Yellow River, Dari County	Yellow River
43	*T. anterodorsalis*	NWIPB1506001		30	Jinsha River, Gonjo County	Yangtze River
44	*T. markehenensis*	NWIPB0907001		31	Dadu River, Danba County	Yangtze River
45	*T*. *strauchii*	NWIPB1305218		32	Kaiken River, Qitai County	Junggar River
46	*T. scleroptera*	NWIPB1250405		33	Yellow River, Guide County	Yellow River
47	*T*. *scleroptera*	NWIPB1308005		34	Lake Qinghai	Lake Qinghai
48	*T*. *orientalis*	NWIPB1006025		35	Tiangeli River, Dulan County	Qaidam River
49	*T. rosa*	20,160,523,001		36	Cave Rosa, Wulong District, Chongqing City	Cave Rosa

Genomic DNA was extracted from the fin or muscle using the standard 3-step phenol-chloroform method [19]. Sequences from two mitochondrial loci (mtDNA: cytochrome *b*, Cyt *b*; 16S ribosomal RNA, 16S) and exon regions of five widely used single-copy nuclear loci (nuDNA: early growth response protein 2B (EGR2B), interphotoreceptor retinoid-binding protein (IRBP), myosin heavy polypeptide 6 (myh6), recombination activating gene 1 (RAG1), and rhodopsin (RH1)) were amplified and sequenced. The primer pairs and PCR conditions are shown in Additional file 2: Table S1. The amplified fragments were sequenced from both ends using an ABI PRISM 3700 sequencing system with the PerkinElmer BigDye DNA Sequencing Kit according to the manufacturer’s protocol, with the same primers used for PCR (Beijing Tianyi Huiyuan Bioscience and Technology Incorporation, Beijing, China).

Sequences were assembled in SeqMan (DNASTAR, Madison, WI, USA), and then aligned using CLUSTAL W [20] in MEGA v6.0 [21] with default parameters. The accuracy was checked manually. Heterozygous sites were resolved using appropriate International Union of Pure and Applied Chemistry (IUPAC) ambiguity codes. All sequences were deposited in GenBank, and accession numbers are listed in Additional file 2: Table S2.

### Multilocus gene tree reconstruction

Because of the linked constraint, Cyt *b* and 16S were concatenated as one mitochondrial locus. We analyzed the phylogenetic relationships of these *Triplophysa* species using maximum likelihood (ML) and Bayesian inference (BI) methods. Gene trees for the mitochondrial locus and the concatenation of the nuclear loci were inferred separately. The optimal evolutionary models for each locus (Additional file 2: Table S3) were identified by PartitionFinder 1.1.1 [22] based on the Bayesian information criterion. The ML analyses were implemented in RAxML v8.2.9 [23] using the GTRGAMMA model under the optimal partitioning scheme (Additional file 2: Table S3). Statistical supports for major nodes were estimated from 1000 bootstrap replicates. The BI analyses were performed in MrBayes v3.2.2 [24]. The posterior distributions from BI were obtained by Markov Chain Monte Carlo (MCMC) analyses with one cold chain and three heated chains using the optimal partitioning strategy as above. The samples of the trees and the parameters were drawn every 5000 steps from a total of 50,000,000 MCMC generations, and the first 25% samples were discarded as burn-in. The convergence was judged with the average standard deviation of split frequencies (< 0.01) and the effective sample size (ESS) values (> 200). Both the ML and BI analyses were implemented in the CIPRES Science Gateway (http://www.phylo.org/index.php). To assess the prominent discrepancy between mitochondrial and nuclear topologies, we performed the approximately unbiased (AU) test [25] using the CONSEL package [26].

### Genealogical sorting index

According to the topology of the mtDNA, we classified *T. stoliczkae* into 4 groups (*T. stoliczkae*-1 to 4). The genealogical sorting index (*gsi*) [27] evaluated the degree of genealogical exclusivity for labeled groups and quantified the genealogical concordance between mtDNA and nuDNA. The normalized *gsi* values ranged from 0 (not monophyletic or clade structure absolutely rejected the group labels) to 1 (monophyletic or clade structure perfectly agrees on the group labels) [28]. The *gsi* analyses were run on each locus trees and combined loci trees. We retained the last 500 rooted trees produced from the BI for the *gsi* analyses. The statistical significance of the *gsi* analyses was tested for all trees using 1000 permutations. All analyses were completed using R package genealogicalSorting ver. 0.92 [29].

### Species tree reconstruction and divergence time

Conspicuous mitonuclear discordances were detected (AU test, *P* < 0.001), but the nuclear results were more in line with the morphological data. In view of the potential gene flows and the disproportionately strong influence from mtDNA [30, 31], we used only the nuclear datasets to infer the species trees and date of divergence. The species designation was based on the analyses above. We treated each group as a potential species, e.g., all individuals of *T. stoliczkae*-1 were treated as species *T. stoliczkae*-1.

The *BEAST [32] species tree analysis and time estimation were simultaneously conducted in BEAST v1.8.0 [33]. The substitution models for each locus were set according to the results from PartitionFinder 1.1.1 [22] (Additional file 2: Table S3). We chose the Yule process as the species trees prior, and a piecewise linear population size with a constant root as the population size prior. A lognormal relaxed molecular clock with an uncorrelated gamma distribution was used. As no fossil record of *Triplophysa* fishes was available, we used one secondary calibration estimated from a previous study [34] to calibrate divergence time, constraining the time of the most recent common ancestor (TMRCA) of *Triplophysa strauchii* in the Ili River and Junggar River to 2.40–3.44 Ma. An accurately dated geological event, the ‘Gonghe movement,’ occurred ca. 0.15 Ma, which caused the separation of the Yellow River and Lake Qinghai [35]. Therefore, we constrained the TMRCA of *Triplophysa scleroptera* in Lake Qinghai and the Yellow River to 0.15 Ma. The effectiveness of these two time-calibration priors was validated in previous studies [15, 36]. We ran four independent runs for 500 million generations with a sampling frequency of every 5000 generations. The convergence of the MCMC posterior parameters was established by an effective sample size (ESS) (> 200) in Tracer 1.5 [37]. Posterior trees from the four runs were combined after removing the first 10% as burn-in in LogCombiner v1.8.0 [33]. The maximum credibility tree was created in TreeAnnotator v1.8.0 [33].

In addition, we also used STAR [38] and MP-EST [39] methods to infer species trees. These two methods allow missing taxa in some gene trees. We performed BI analyses for each nuclear locus using the best substitution model (Additional file 2: Table S3), setting 50,000,000 MCMC generations runs and sampling every 5000 generations. After removing the first 25% trees as burn-in, we randomly sampled 1000 trees for each locus. These trees were used in the Species Tree Analysis (STRAW) online server (http://bioinformatics.publichealth.uga.edu/SpeciesTreeAnalysis/index.php) to conduct the STAR and MP-EST analyses. We ran each twice to ensure congruence.

### Reconstructing ancestral state

*Triplophysa stoliczkae* is a scrape-feeding fish and has apomorphic character states: broadened sharp lower jaw, screw-like intestine, and degenerated PCAB [11, 12] (Additional file 1: Figure S2). In the genus *Triplophysa*, nearly all the scrape-feeding species share this combination of characteristics, which implies that scrape-feeding fish is a specific group in *Triplophysa*. To infer the occurrence of scrape-feeding fish, especially the occurrence of “*T. stoliczkae*”, we assigned *Triplophysa* into three simplified types: *T. stoliczkae* type, other scrape-feeding type, and normal-feeding (predatory) type. The assignment was based on the literatures [11, 12] and our field surveys.

The evolutionary trajectories of these types were reconstructed by the ML approach in Mesquite 2.75 [40]. Considering topological uncertainty, we used 10,000 *BEAST species trees (sampled the combined trees file and conducted it twice to confirm congruence) for this inference. The Markov *K*-state 1 model was selected, and the character transitions were considered to be disordered and reversible. The transition number between character states was calculated using the “summarize state changes over trees”.

### Morphospace construction and convergence estimates

To examine phenotypic changes in the genus *Triplophysa*, we conducted morphological measurements and combined them with our species tree estimate. Due to typical cave-dwelling characteristics (e.g., eyes disappeared) and the lack of specimens, we removed *T. rosa* from the analyses. We measured 14 widely used morphometric characteristics, which can fully reflect the morphology of a species, from 176 individuals across 26 species (details in Additional file 3: Table S4). As per Blom et al. [41], we calculated residual values of each trait variation from phylogenetically corrected regressions using log-transformed traits against log-transformed standard length in R package Phytools (function *phyl.resid*; [42]). To eliminate multicollinearity and reduce the multidimensionality of the data, we conducted a phylogenetically corrected principal component analysis (pPCA) using the residuals of these traits. The scores on the main PC axes were retained and adopted as trait input for subsequent analyses. We then projected the significant PCs on a phylomorphospace (function *phylomorphospace*; [42]) to evaluate interspecific phenotypic changes.

To further quantify the convergence of “*T. stoliczkae*”, we calculated the amount of independently evolved similarity within PC scores by both distance-based (*C*_1–4_) and frequency-based (*C*_5_) measures of convergence in the R package *convevol* [43]. We performed 500 simulations using Brownian motion along the phylogeny to assess the significance of the observed *C* values.

## Results

### Sequence information

We obtained 1140 bp of Cyt *b* and 1025 bp of 16S rRNA. The five nuclear loci for EGR2B, IRBP, myh6, RAG1, and RH1 were 754 bp, 657 bp, 713 bp, 1505 bp, and 712 bp in final aligned length, respectively. After concatenation, the mtDNA was 2165 bp in length and consisted of 511 parsimony-informative sites. The nuDNA was 4341 bp in length and consisted of 317 parsimony-informative sites.

### Phylogenetic analyses

**Fig. 2:**
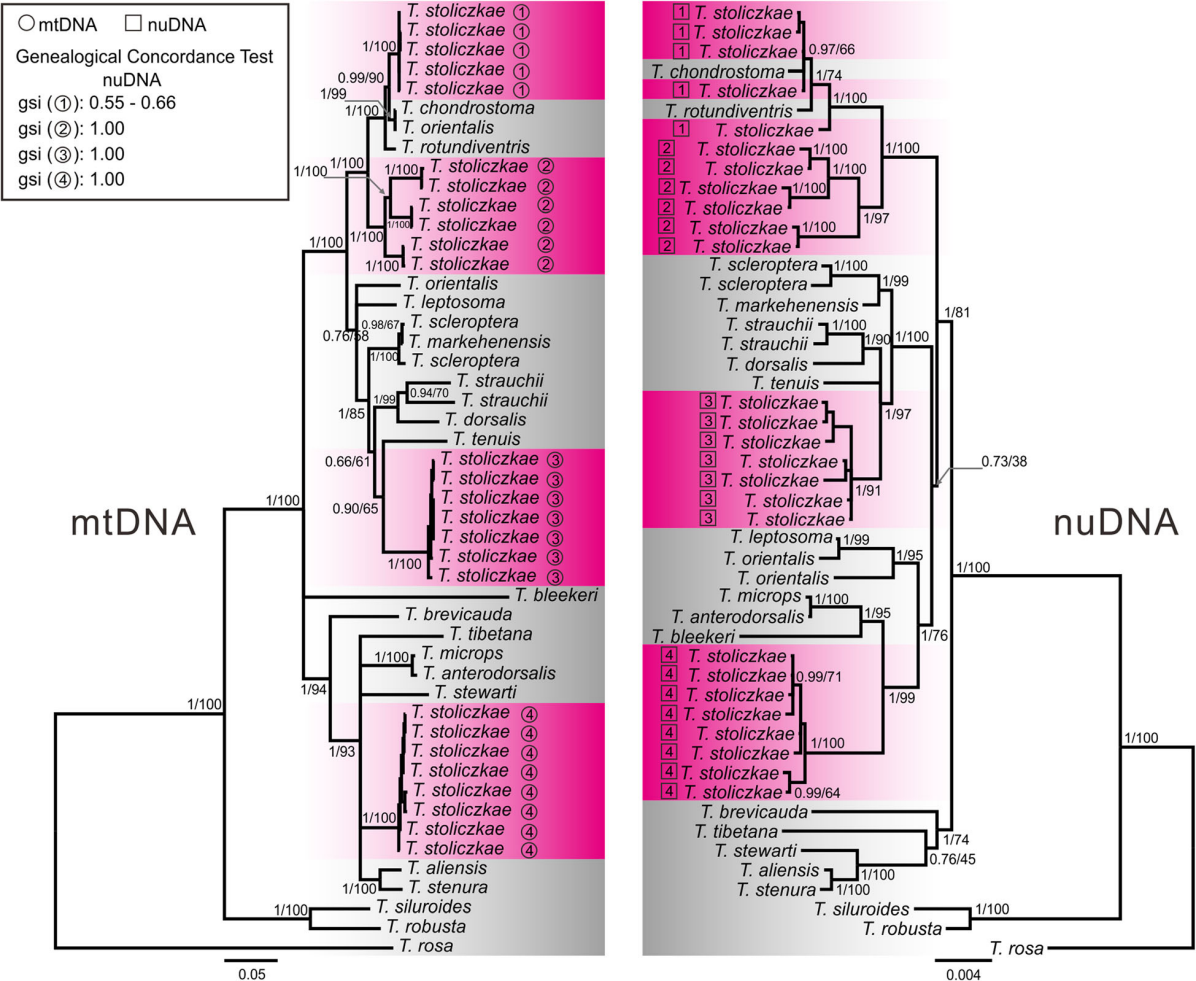
Phylogenetic reconstructions based on mtDNA (left) and nuDNA (right). Both trees are generated from a Bayesian inference. Values on the key nodes indicate the posterior probabilities (PPs) obtained in the Bayesian inference and bootstrap support (BS) from the maximum likelihood analyses (PP/BS). The background colors are denoted as those in Fig. 1. *Triplophysa stoliczkae* individuals are labeled into 4 groups corresponding to the mtDNA clades, with circles for mtDNA and squares for nuDNA. The genealogical concordance between mtDNA and nuDNA trees are evaluated by the genealogical sorting index (*gsi*) values listed in the box

The ML and BI analyses yielded generally congruent phylogenies. In the mtDNA results (Fig. 2), *T. stoliczkae* clustered into four distinct clades, all of which were monophyletic with high support values (PP = 1; BS = 100). Hence, we labeled *T. stoliczkae* into four groups (*T. stoliczkae*-1 to *T. stoliczkae*-4). Specifically, *T. stoliczkae*-1 consisted of individuals from the Yangtze River. *T. stoliczkae*-2 comprised individuals from the Yellow River, the Shiyang River and the Heihe River. *T. stoliczkae*-3 contained individuals from the Ili River. *T. stoliczkae*-4 included individuals from the Tarim River, the Indus River, and the Ganges River.

The results based on nuDNA recovered similar topologies for *T. stoliczkae* as those based on mtDNA (Fig. 2). *T. stoliczkae*-2, *T. stoliczkae*-3, *T. stoliczkae*-4 were distinct monophyletic clades with high support values (PP = 1; BS ranged from 91 to 100), while *T. stoliczkae*-1 formed a clade with two other *Triplophysa* species (PP = 1; BS = 100), *T. chondrostoma* and *T. rotundiventris*. The conservative AU test detected a significant conflict between mtDNA and nuDNA topologies (*P* < 0.001). The conflict was mainly due to the relationships among other *Triplophysa* species.

### Genealogical sorting indices

We set an accepted priori value of *gsi* > 0.80. Four labeled *Triplophysa stoliczkae* groups reached monophyly in the mtDNA test (Table 2). The nuDNA results indicated significant monophyly in the groups except for *T. stoliczkae*-1 (*gsi* = 0.64). Individual nuclear loci suggested moderate genealogical divergence in *T. stoliczkae*-1 (*gsi* ranged from 0.66 to 0.73). *T. stoliczkae*-2 reached or was close to reaching monophyly (*gsi* ranged from 0.78 to 1.00). *T. stoliczkae*-3 reached monophyly at the EGR2B locus (*gsi* = 0.99), and moderate genealogical divergence at other nuclear loci (*gsi* ranged from 0.45 to 0.73). *T. stoliczkae*-4 recovered robust genealogical exclusivity at all nuclear loci (*gsi* ranged from 0.90 to 1.00). All analyses rejected the monophyly of *T. stoliczkae* (*gsi* ranged from 0.35 to 0.56). The discordance between mtDNA and nuDNA in *T. stoliczkae*-1 was quantified by relatively low *gsi* values (from 0.55 to 0.66, Fig. 2).

**Table 2:** Genealogical sorting index (*gsi*) and probability values of *Triplophysa stoliczkae* and the four labeled groups

Gene	*T. stoliczkae*-1		*T. stoliczkae*-2		*T. stoliczkae*-3		*T. stoliczkae*-4		*T. stoliczkae*	
	*gsi*_*T*_	*P*	*gsi*_*T*_	*P*	*gsi*_*T*_	*P*	*gsi*_*T*_	*P*	*gsi*_*T*_	*P*
mtDNA	1	< 0.001	1	< 0.001	1	< 0.001	1	< 0.001	0.349	< 0.001
nuDNA	0.637	< 0.001	1	< 0.001	1	< 0.001	1	< 0.001	0.559	< 0.001
RH1	0.686	< 0.001	0.782	< 0.001	0.452	< 0.001	1	< 0.001	0.535	< 0.001
myh6	0.662	< 0.001	0.793	< 0.001	0.634	< 0.001	1	< 0.001	0.484	< 0.001
IRBP	0.673	< 0.001	0.815	< 0.001	0.605	< 0.001	1	< 0.001	0.468	< 0.001
RAG1	0.73	< 0.001	1	< 0.001	0.726	< 0.001	1	< 0.001	0.368	0.002
EGR2B	0.684	< 0.001	0.999	< 0.001	0.99	< 0.001	0.902	< 0.001	0.448	< 0.001

*gsi*_*T*_ is derived from the last 500 trees in the Bayesian inference

### Species trees and divergence times

**Fig. 3:**
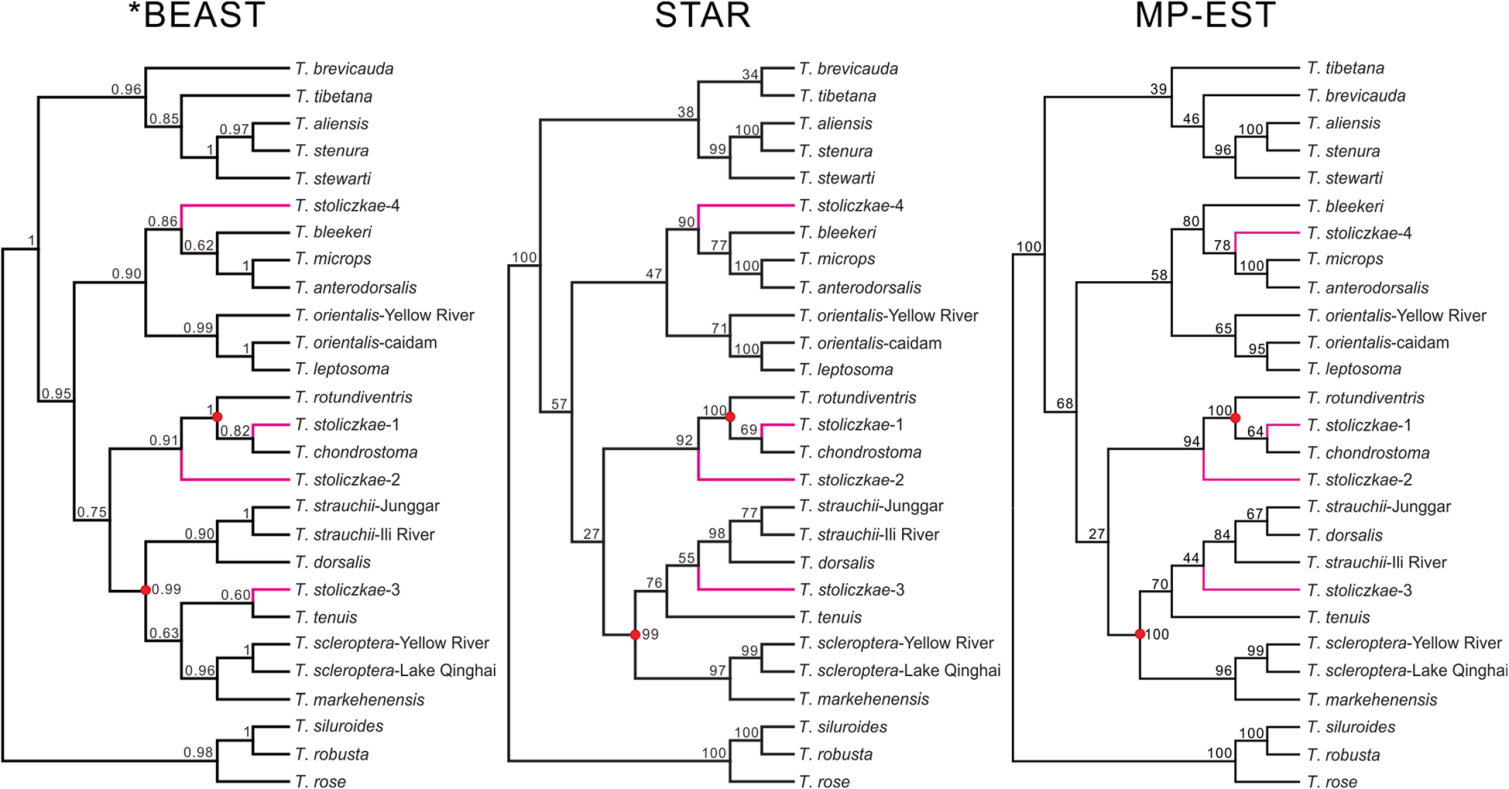
Species tree reconstructions of *Triplophysa* using three different methods based on five nuclear loci. Trees are maximum clade credibility cladograms (w/o branch lengths). Numbers on the nodes denote the posterior support for *BEAST and bootstrap support for STAR and MP-EST.

**Fig. 4:**
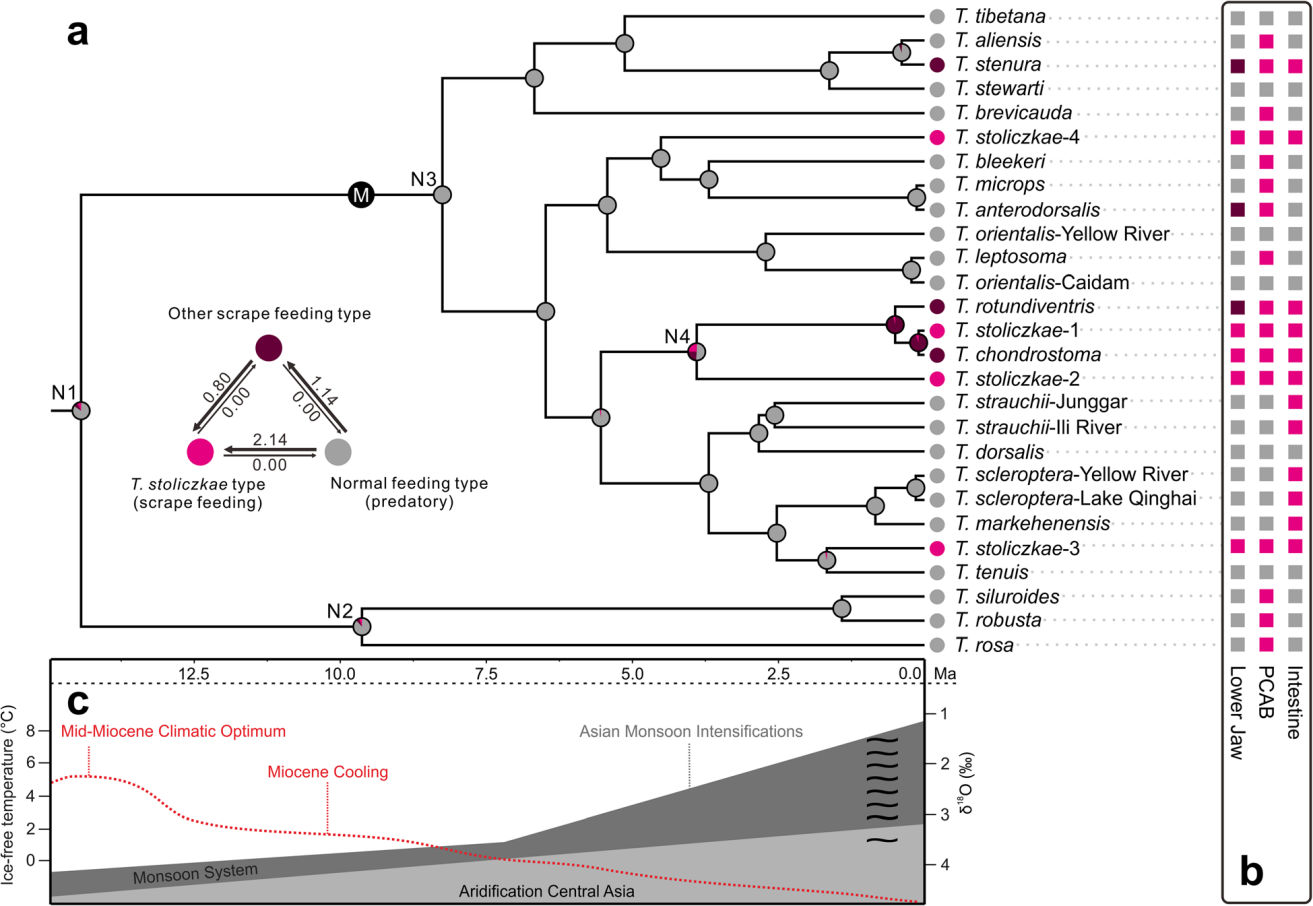
The evolutionary trajectory of the three simplified types in *Triplophysa*. **a** Time-calibrated Bayesian species tree from the *BEAST analysis. Circles on the tips indicate three simplified types, and pie charts at ancestral nodes represent their relative ML supports. The triangle with arrow indicates the direction with unequivocal transition numbers (average). N1-N4 denote four nodes. **b** Three characteristics of *Triplophysa*. Lower jaw: broadened, sharp and uncovered by lips (rose red); spoon-like, sharp and uncovered by lips (black red); spoon-like, blunt and covered by lips (gray). PCAB (posterior chamber of air bladder): developed (gray) and degenerated (rose red). Intestine: screw shape (rose red) and zig-zag shape (gray). Details are depicted in Additional file 1: Figure S5. **c** Climatic events (modified from Favre et al. [17] and Zachos et al. [18]): the red dotted line denotes the major global temperature trends; tildes (∼) represent the Quaternary climate oscillations

The interspecific relationships among the *Triplophysa* are not easily resolved. Although low support values occurred in a certain number of nodes, we noted that the three coalescent methods recovered generally congruent topologies (Fig. 3). The four *T. stoliczkae* groups were nested into distinct lineages, but all had moderate support values with their sibling clades. Nevertheless, *T. stoliczkae*-1 and *T. stoliczkae*-3 were strongly distinct from the other two *T. stoliczkae* groups because of strong support at the base of the clades (red dot in Fig. 3). The TMRCA for clade M was approximately 8.25 Ma (95% highest posterior density (HPD): 4.21–14.36 Ma) (Fig. 4a and Additional file 1: Figure S3). The divergence events between “*T. stoliczkae*” and the other species occurred from approximately 0.10 to 4.51 Ma. The earliest split emerged between *T. stoliczkae*-4 and its sibling group (*c*. 4.51 Ma; 95% HPD: 2.12–8.04 Ma), and the latest split occurred between *T. stoliczkae*-1 and its sibling species (*c*. 0.10 Ma; 95% HPD: 0.00–0.41 Ma).

### Evolution of ancestral state

We considered a state to be reconstructed when the relative likelihood (RL) was greater than 0.87. Although normal-feeding was slightly equivocal (RL = 0.84) for the ancestral (N1) state of *Triplophysa* fish, it was reconstructed at basal nodes N2 and N3 (RL = 0.88 and 0.99, respectively; Fig. 4a). The states of the remaining nodes were well reconstructed except for N4. In the N4 node, the RL for normal-feeding was 0.51, and the RLs of the other two scrape-feeding types were 0.24 and 0.25. The reconstruction analyses indicated that scrape-feeding most likely stemmed from the normal-feeding at least four times and that *T. stoliczkae*-1 further experienced morphological convergence with other “*T. stoliczkae*”-like species (*T. stoliczkae*-3 and *T. stoliczkae*-4 and possibly *T. stoliczkae*-2). The evolutionary tendencies were supported by the ML number of unequivocal transitions (arrows triangle in Fig. 4a).

### Morphometric and *convevol* analyses

**Fig. 5:**
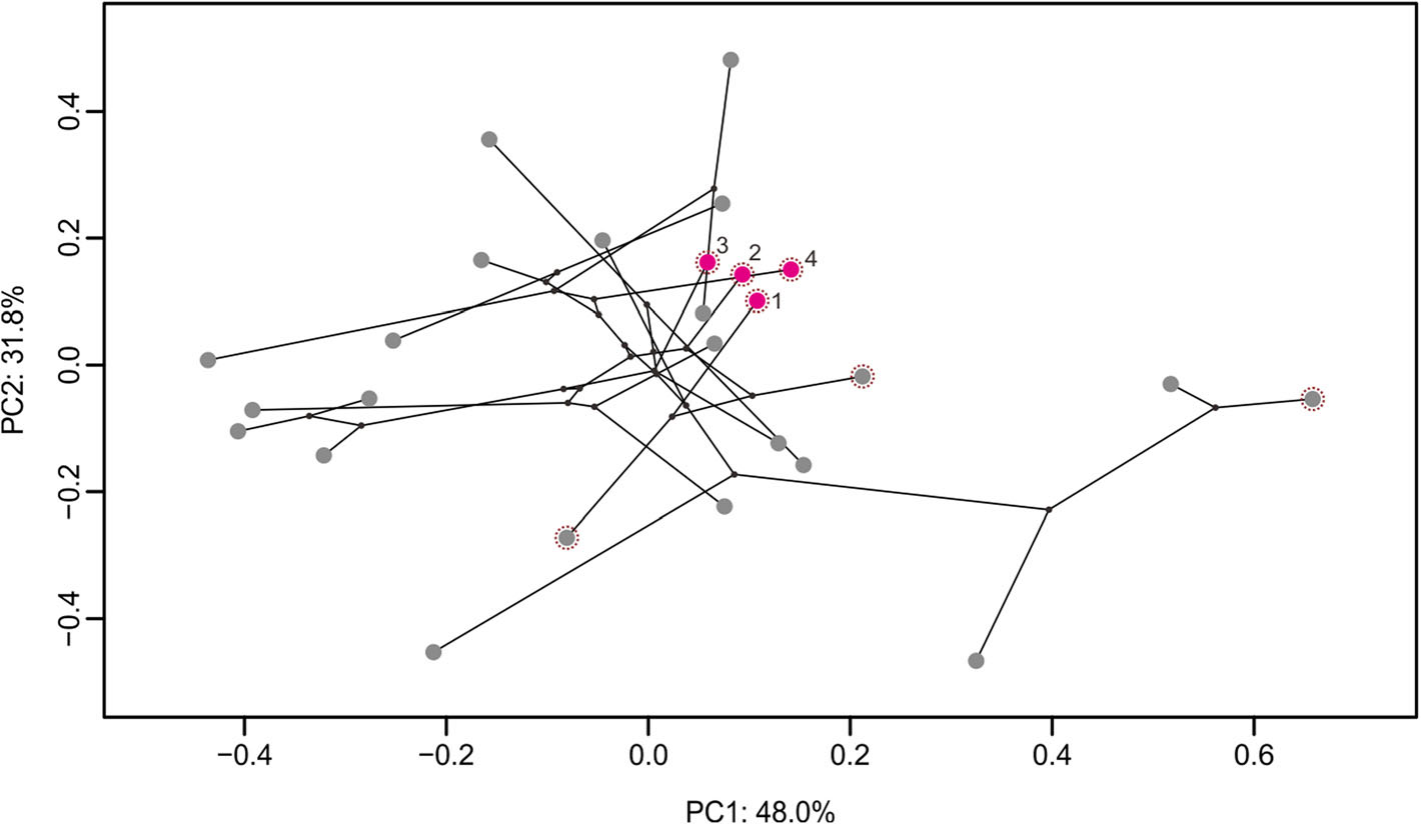
Phylomorphospace plot of *Triplophysa*, superimposing the branching patterns of the phylogeny (black lines) onto the first two PC axes. Each dot represents a species phenotypic value (rose red and gray) or a hypothesized ancestral phenotype (black). Rose-red dots denote *T. stoliczkae*-1 to *T. stoliczkae*-4 and gray dots indicate other *Triplophysa* species. Scrape-feeding species are highlighted with a dotted circle

*Triplophysa* displayed considerable morphological diversity (Fig. 5). The pPCA yielded two significant axes responsible for nearly 80% of the variance within the morphological dataset. PC1 and PC2 explained approximately 48 and 31.8% of the variance, respectively. Four lineages of “*T. stoliczkae*” clustered together in morphospace and represented a morphological type different from the others (Fig. 5 and Additional file 1: Figure S4). Moreover, these four lineages showed closer phenotypic distances than that of their ancestors.

Both distance-based and frequency-based measures found significantly convergent among the “*T. stoliczkae*” lineages (*P* ≤ 0.012; Table 3). Analyses revealed a *C*_1_ of 0.77, indicating that evolution has closed 77% of the distance among “*T. stoliczkae*” lineages. Moreover, convergence accounted for 4% of the total evolution in the smallest clade containing four “*T. stoliczkae*” lineages (*C*_4_ = 0.04). These four “*T. stoliczkae*” lineages were also found to be significantly convergent using the frequency-based measure (*C*_5_ = 4; Additional file 1: Figure S4).

**Table 3:** Convergence test results

Convergence metric	Estimate	*P*
*C*_1_	0.77	< 0.001
*C*_2_	0.19	< 0.001
*C*_3_	0.03	0.012
*C*_4_	0.04	0.012
*C*_5_	4	0.006

## Discussion

### Convergent evolution in *Triplophysa stoliczkae*

Conspicuous mitonuclear discordances (Fig. 2; AU test *P* < 0.001) suggest that the genus *Triplophysa* presumably has experienced extensive introgression and/or incomplete lineage sorting (ILS), which may also account for the weak statistical support for most branches in the coalescent analyses (Fig. 3). Introgressive events always interfere with a coalescent analysis and cause low statistical support [31, 36], which has been reported in some *Triplophysa* species [36]. Moreover, although the coalescent analysis can accommodate the ILS, insufficient information in nuclear loci will also cause moderate support values. Both introgression and ILS generally cause counterintuitive phylogenetic relationships in intra- and interspecies [31, 44]. These two factors can explain the moderate coalescent support for the four *T. stoliczkae* lineages but not the source of the nonmonophyly of *T. stoliczkae* for two main reasons: (1) introgression has striking impacts on the mtDNA tree, while ILS is prone to give misleading nuDNA results [30, 45]. In our case, the discordances were mainly due to the relationships among other *Triplophysa* species and not those within the *T. stoliczkae* (*T. stoliczkae*-1 see below). Both the mtDNA and the nuDNA trees indicate that “*T. stoliczkae*” includes four distinct species, and mtDNA results described a clearer and more reasonable topology (Fig. 2). (2) All three species tree methods can accommodate the ILS [32, 38, 39]. However, none of these methods recovered the monophyly of *T. stoliczkae* (Fig. 3). Hence, we reject the scenario that *T. stoliczkae* originated only once from a single ancestor. *T. stoliczkae*-1 forms a monophyletic group with *T. chondrostoma* and *T. rotundiventris* in the nuDNA tree (Fig. 2), which may result from the ILS (not introgression). Due to the lower sorting rates of nuDNA, the ILS is more prone to influence nuDNA than mtDNA results, especially among closely related species [30, 45]. Accommodated the ILS, coalescent analyses recover congruent interspecific relationships with the mtDNA tree for these three species (Figs. 2 and 3). In conclusion, *T. stoliczkae* consists of four distinct lineages that are not closest relatives.

The ancestral state reconstruction analysis identified the independent innovations of the “*T. stoliczkae*” type in distinct lineages (Fig. 4a). Morphometric and *convevol* analyses yielded significant phenotypic convergence between the “*T. stoliczkae*” lineages (Fig. 5; Table 3). These lines of evidence strongly support that *T. stoliczkae* was the product of convergent evolution. The occurrences of “*T. stoliczkae*” (*c.* 4.51–0.10 Ma) were just in the period of intense tectonic movements and dramatic climatic change on the QTP and its peripheral regions [46, 47]. In addition, three typical characteristics of *Triplophysa* occurred “randomly” in the phylogeny (Fig. 4b). The typical characteristic combination associated with scrape feeding behavior indicated ecological adaptation. Therefore, we speculate that these historical upheavals created similar ecological conditions, which led to the repeated and directed evolution in “*T. stoliczkae*”.

The TMRCA of clade M occurred at approximately 8.25 Ma (Fig. 4a). Geological studies have suggested that the large-scale intense uplift of QTP began at approximately 8 Ma [46, 47], from which the macro-environment became more severe on the QTP (Fig. 4c) [17, 18]. Palynological studies have indicated that a marked ecological shift towards a drier climate occurred *c.* 4.5 Ma on the QTP [48] when the earliest split between “*T. stoliczkae*” and its sibling group was occurring (Fig. 4a). Therefore, it was reasonable to believe that the intense tectonic movements caused radiation of the species. In addition, due to the uplift and the increasingly harsh climate, the species on the QTP experienced high levels of extinction. Rivers and lakes became increasingly oligotrophic. To cope with the growing food shortage, some populations of *Triplophysa* fishes had to feed on algae with low-nutrient contents on the surfaces of stones and mud [12]. Eventually, the scrape-feeding behavior was fixed in certain populations that had been engaging in normal-feeding (predatory) behavior. A sharp lower jaw and long intestine were required for scrape-feeding fish to consume the algae [12]. The long intestine formed a screw shape. Moreover, scrape-feeding fish lay on stone, scraping with the force produced by the body’s swing, without vertical movement. A degenerated PCAB is more suitable for scrape-feeding fish to maintain a balanced body during feeding. Therefore, these typical features are found in all scrape-feeding *Triplophysa* fishes. Furthermore, without vertical movement, in comparison to an inflated and lateral opening of an air bladder, a moderate and lateral closed bony capsule of an air bladder is more suitable for scrape-feeding fish because of the reduced demand for sensing water pressure changes [12]. Both a thick caudal peduncle and emarginate caudal fin are also suitable for scrape-feeding behavior [11, 12]. The optimization of morphological structure strengthens convergent evolution (such as *T. stoliczkae*-1). The innovation of scrape feeding, coupled with associated morphological adaptations, led to convergent evolution in *T. stoliczkae*.

### Implications for taxonomy

*Triplophysa stoliczkae* is the most controversial species in the genus *Triplophysa* [11–13]. Because of the cursory morphological description, many species (*T. tibetana*, *T. brevicauda*, *T. leptosoma*, *T. stenura*, *T. tenuicauda*, *T. stewarti*) were once mistaken as *T. stoliczkae* [11, 12, 49, 50]. Currently, we refer to the revised classification [11, 12], which still considers *T. stoliczkae* as a single species spreading throughout the QTP. However, our results show that *T. stoliczkae* should be reclassified into four separate species. In addition, previous studies have also described subtle morphological differences among different “*T. stoliczkae*” populations [11, 12]. *T. stoliczkae* was first described from Lake Tsumuriri in the upper Indus River system [51]. Therefore, we suggest retaining the name *T. stoliczkae* for populations in the Indus River system, as well as the populations in the Ganges River and the Tarim River. *Triplophysa dorsonotatus* has been described from the Kunes River (Ili River system) but was later synonymized with *T. stoliczkae* [11, 12, 52]. We suggest recommissioning the name *T. dorsonotatus* for the “*T. stoliczkae*”-like species from the Ili River system. The population in the Yangtze River is a distinct species and populations in the Yellow River, the Shiyang River, and the Heihe River are from another separate species. Further descriptions are needed to redefine the two species. Our work, with a limited number of individuals per species, represents a conservative study. Additional samples might reveal additional *T. stoliczkae* lineages.

## Conclusions

In summary, *T. stoliczkae* presents a striking case of morphological convergence. The harsh climate that has occurred since the Pliocene forced some populations of the *Triplophysa* fishes to evolve from predatory feeding to scrape feeding. This change in feeding behavior together with associated morphological adaptations caused morphological convergence and created the observed widespread “*T. stoliczkae*” species. A further taxonomic revision of *T. stoliczkae* is advisable.

## Additional files

Additional file 1: **Figure S1.** Photo of the study species, *Triplophysa stoliczkae* in Lake Pangong, which is part of the Indus River system. **Figure S2.** *Triplophysa stoliczkae*. a Lateral view. b Ventral view (mouth). c Air bladder. d Intestine. c–d Data from Wu and Wu [1]. **Figure S3.** A time-calibrated Bayesian species tree from the *BEAST analysis. Numbers near the nodes indicate mean values (95% highest posterior density) of divergence time (Ma). **Figure S4.** A frequency-based measure of convergence. A phylomorphospace of *Triplophysa* based on the first two PC axes (pPCA, Fig. 4a) is shown. Dots in the circle represent the four focal taxa of interest (*T. stoliczkae*-1 to *T. stoliczkae*-4). The purple ellipse indicates the phenotypic space of these focal taxa. Red arrows indicate four lineages that cross into this ellipse. **Figure S5.** a–b Intestine: a. screw shape (*Triplophysa stoliczkae*), b. zig-zag shape (*T. siluroides*). c–e Air bladder: c. PCAB (posterior chamber of air bladder) completely degenerated (*T. stoliczkae*), d. PCAB degenerated and almost invisible to the eye (*T. robusta*), e. PCAB developed (*T. tibetana*). f–h Lower jaw: f. broadened, sharp and uncovered (*T. stoliczkae*), g. spoon-like, sharp and uncovered (*T. stenura*) and h. spoon-like, blunt and covered by lips (*T. siluroides*). Data a–d from Wu and Wu [1]. Data e from Zhu [2]. Data g–h from Wu and Wu [1]. (DOCX 872 kb) (DOCX 871 kb)

Additional file 2: **Table S1.** Primers used in this study and PCR conditions. **Table S2.** GenBank accession numbers for the specimens included in this study. Sample ID corresponds to Fig. 1. **Table S3.** The optimal partitioning scheme and substitution models for each locus. (DOCX 45 kb) (DOCX 44 kb)

Additional file 3: **Table S4.** 14 morphometric characters used in the morphospace construction. Including standard length (SL), preanal length (PreanalL), preanus length (PreanusL), prepelvic length (PrepelvicL), caudal peduncle length (CPL), head length (HeadL), prepectoral length (PrepectoralL), snout length (SnoutL), postorbital length (PostorbitalL), interorbital width (InterorbitalW), eye diameter (EyeD), head depth (HeadD), body depth (BodyD), and caudal peduncle depth (CPD). (CSV 15 kb) (CSV 14 kb)

## Data Availability

All nucleotide sequences are available in GenBank [MG725380-MG725614, MG735502-MG735539].
