# Supplementary material for: Multiple convergent events created a nominal widespread species: Triplophysa stoliczkae (Steindachner, 1866) (Cobitoidea: Nemacheilidae)
Source: BMC Evol Biol. 2019 Sep 4;19:177. doi: 10.1186/s12862-019-1503-3 (PMC6724303; doi:10.1186/s12862-019-1503-3)
Supplement: Supplementary file 1 — Figure S1. Photo of the study species, Triplophysa stoliczkae in Lake Pangong, which is part of the Indus River system. Figure S2. Triplophysa stoliczkae. a Lateral view. b Ventral view (mouth). c Air bladder. d Intestine. c–d Data from Wu and Wu [1]. Figure S3. A time-calibrated Bayesian species tree from the *BEAST analysis. Numbers near the nodes indicate mean values (95% highest posterior density) of divergence time (Ma). Figure S4. A frequency-based measure of convergence. A phylomorphospace of Triplophysa based on the first two PC axes (pPCA, Fig. 4a) is shown. Dots in the circle represent the four focal taxa of interest (T. stoliczkae-1 to T. stoliczkae-4). The purple ellipse indicates the phenotypic space of these focal taxa. Red arrows indicate four lineages that cross into this ellipse. Figure S5. a–b Intestine: a. screw shape (Triplophysa stoliczkae), b. zig-zag shape (T. siluroides). c–e Air bladder: c. PCAB (posterior chamber of air bladder) completely degenerated (T. stoliczkae), d. PCAB degenerated and almost invisible to the eye (T. robusta), e. PCAB developed (T. tibetana). f–h Lower jaw: f. broadened, sharp and uncovered (T. stoliczkae), g. spoon-like, sharp and uncovered (T. stenura) and h. spoon-like, blunt and covered by lips (T. siluroides). Data a–d from Wu and Wu [1]. Data e from Zhu [2]. Data g–h from Wu and Wu [1]. (DOCX 872 kb) (DOCX 871 kb) [file 12862_2019_1503_MOESM1_ESM.docx]

**
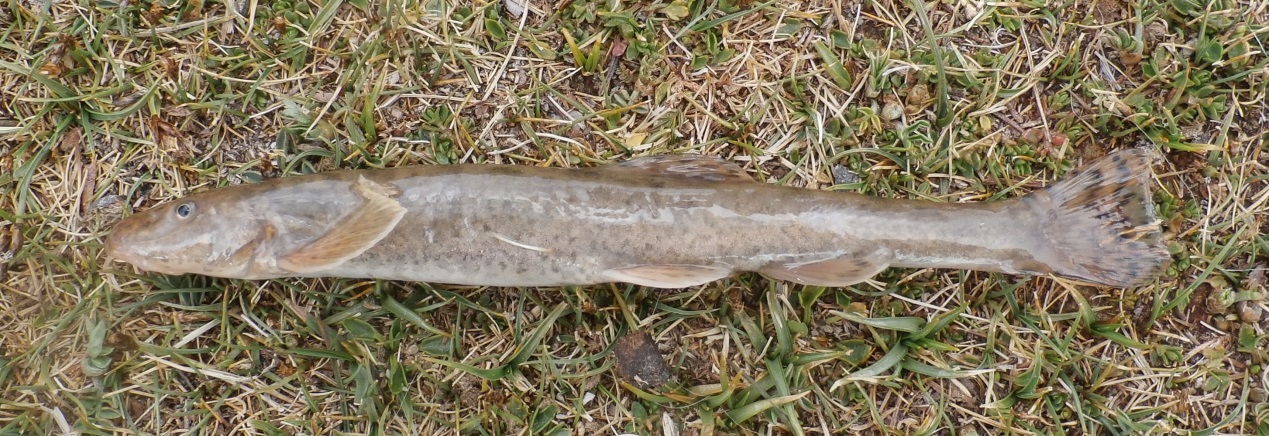
Fig. S1** Living photo about the study species, *Triplophysa stoliczkae* in the Lake Pangong, which is part of the Indus River system.

**
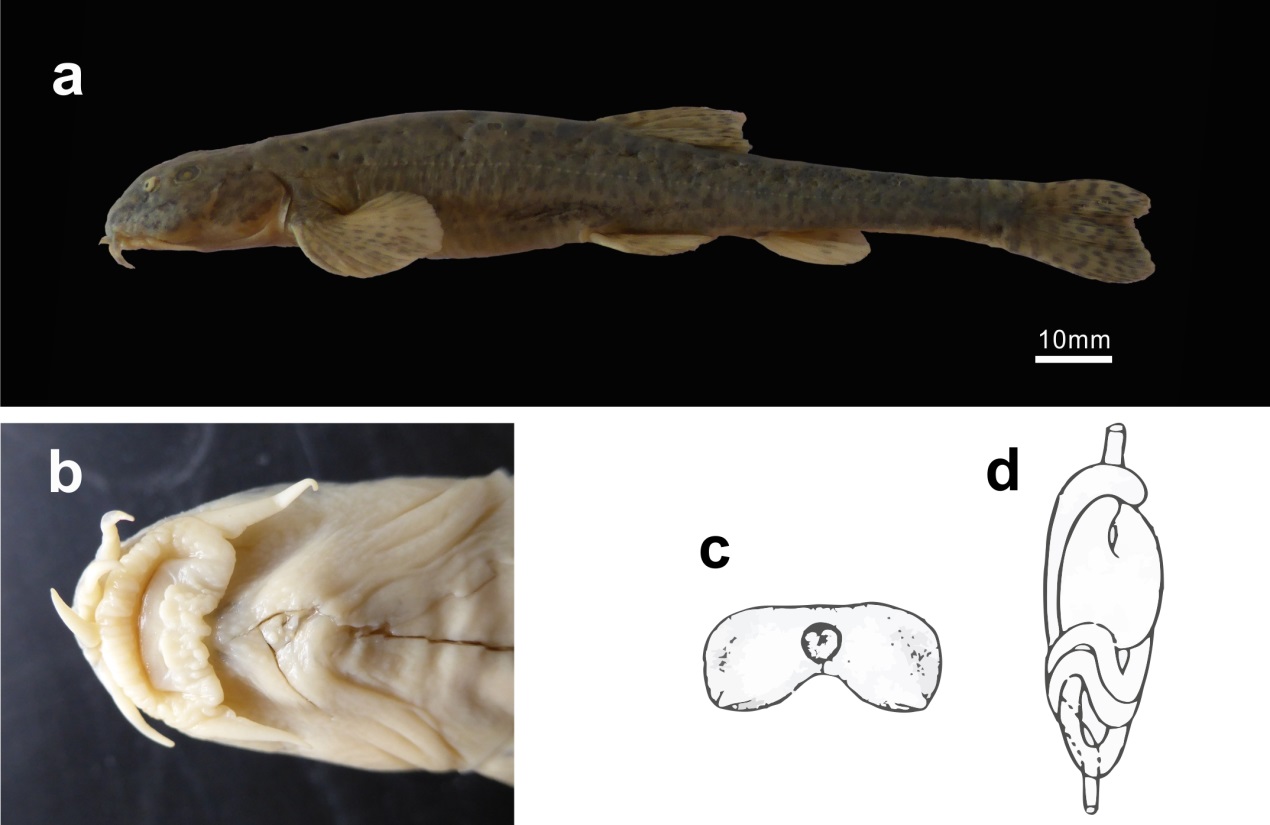
Fig. S2** *Triplophysa stoliczkae*. **a** Lateral view. **b** Ventral view (mouth). **c** Air bladder. **d** Intestine. **c**–**d** Data from Wu and Wu [1].


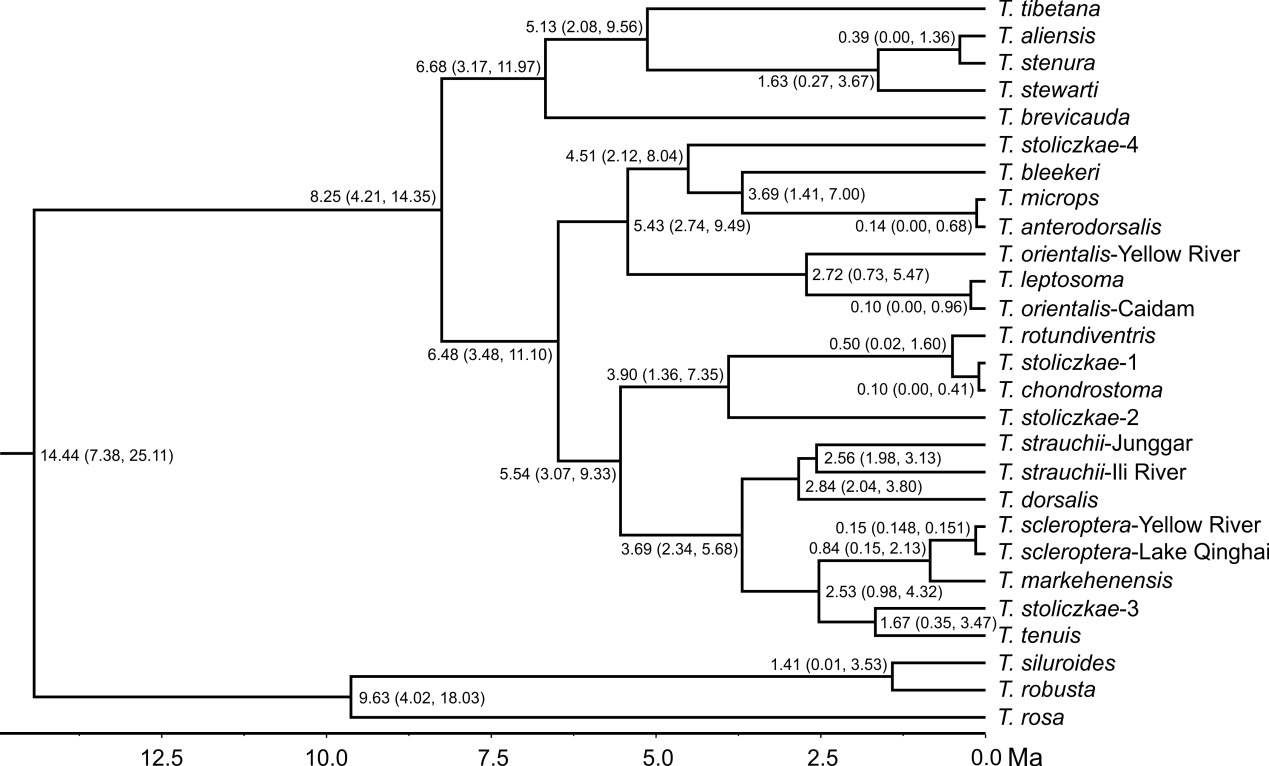
 **Fig. S3** Time-calibrated Bayesian species tree from the *BEAST analysis. Numbers near the nodes indicate mean values (95% highest posterior density) of divergence time (Ma).

**
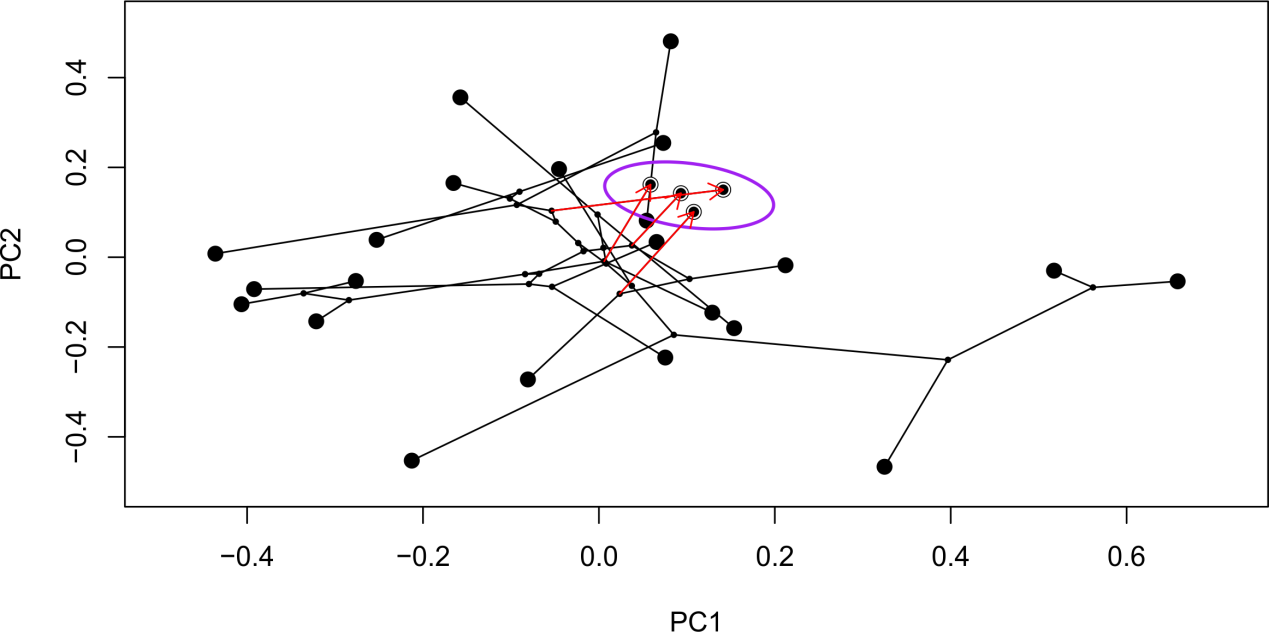
Fig. S4** Frequency-based measure of convergence. A phylomorphospace of *Triplophysa* based on the first two PC axes (pPCA, Fig. 4a) is shown. Dots in the circle represent four focal taxa of interest (*T. stoliczkae*-1 to 4). Purple ellipse indicates the phenotypic space of these focal taxa. Red arrows indicate four lineages that cross into this ellipse. **
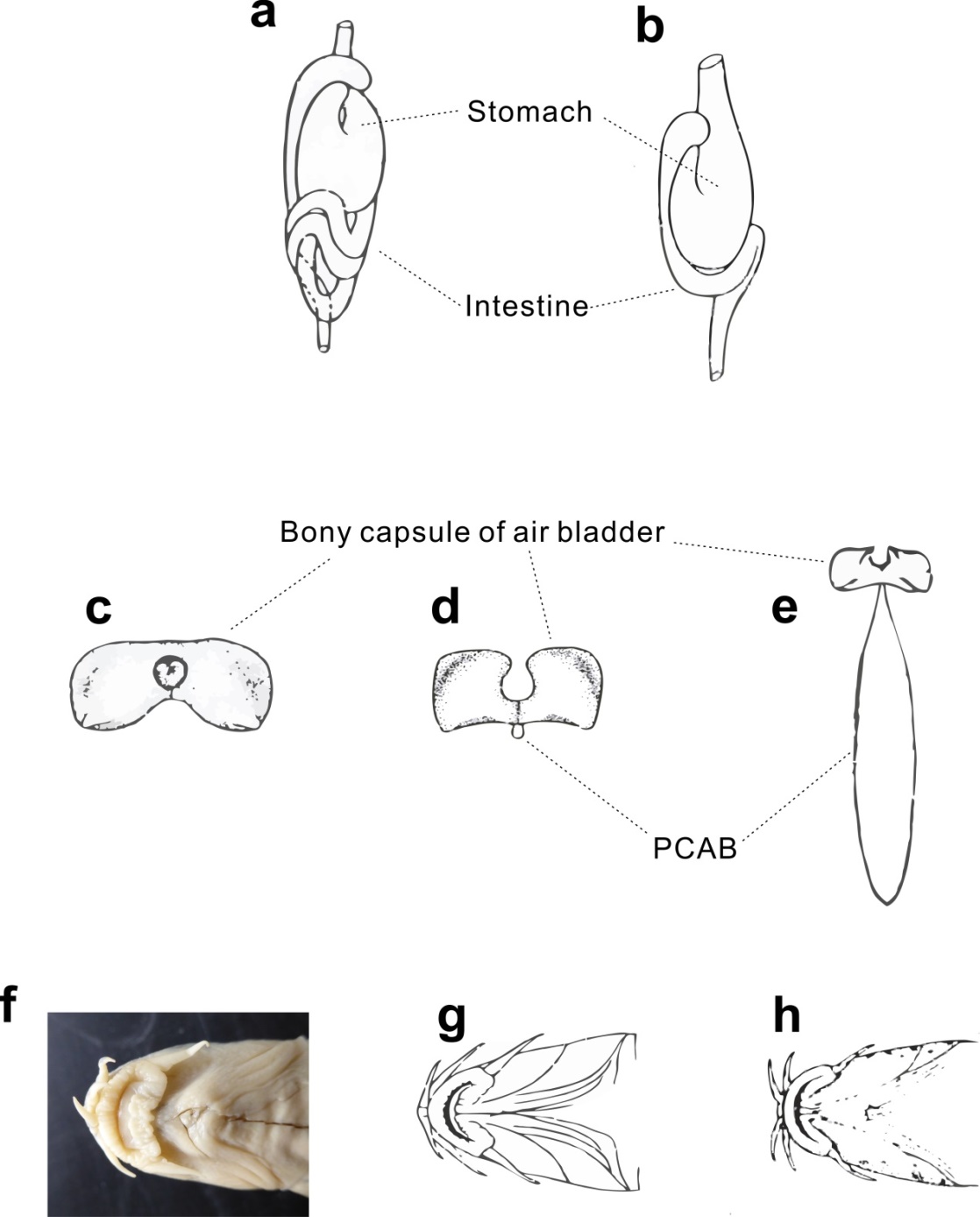
**

**Fig. S5** **a**–**b** Intestine: **a** screw shape (*Triplophysa stoliczkae*), **b** zig-zag shape (*T*. *siluroides*). **c**–**e** Air bladder: **c** PCAB (posterior chamber of air bladder) completely degenerated (*T. stoliczkae*), **d** PCAB degenerated and almost invisible by eyes (*T*. *robusta*), **e** PCAB developed (*T*. *tibetana*). **f**–**h** Lower jaw: **f** broadened, sharp and uncovered (*T. stoliczkae*), **g** spoon-like, sharp and uncovered (*T*. *stenura*) and **h** spoon-like, blunt and covered by lips (*T*. *siluroides*). Data **a**–**d** from Wu and Wu [1]. Data **e** from Zhu [2]. Data **g**–**h** from Wu and Wu [1].

**References**

1. Wu Y, Wu C. The fishes of the Qinghai-Xizang plateau. Chengdu: Sichuan Publishing House of Science and Technology; 1992.

2. Zhu S. The loaches of the subfamily Nemacheilinae in China (Cypriniformes: Cobitidae). Nanjing: Jiangsu Science and Technology Publishing House; 1989.
