# Supplementary material for: Multiple convergent events created a nominal widespread species: Triplophysa stoliczkae (Steindachner, 1866) (Cobitoidea: Nemacheilidae)
Source: BMC Evol Biol. 2019 Sep 4;19:177. doi: 10.1186/s12862-019-1503-3 (PMC6724303; doi:10.1186/s12862-019-1503-3)
Supplement: Supplementary file 2 — Table S1. Primers used in this study and PCR conditions. Table S2. GenBank accession numbers for the specimens included in this study. Sample ID corresponds to Fig. 1. Table S3. The optimal partitioning scheme and substitution models for each locus. (DOCX 45 kb) (DOCX 44 kb) [file 12862_2019_1503_MOESM2_ESM.docx]

**Table S1** Primers used in this study and PCR conditions.

| **Locus** | **primer name** | **Primer sequence** | **Source** | **PCR conditions** |
| --- | --- | --- | --- | --- |
| Cytb | L14724 | GACTTGAAAAACCACCGTTG | Xiao et al.’s [1] | 95°C-5 min, [94°C-30 sec, 52°C-30 sec, 72°C-90 sec]35 loops, 72°C-10 min |
|  | H15915 | CTCCGATCTCCGGATTACAAGAC | Xiao et al.’s [1] |  |
| 16S | 16Sp1F | CTTACACCGAGAARACATC | Li et al.’s [2] | 95°C-5 min, [94°C-30 sec, 52°C-30 sec, 72°C-90 sec]35 loops, 72°C-10 min |
|  | 16Sp1R | CTTAAGCTCCAAAGGGTC | Li et al.’s [2] |  |
| RH1 | RH1F | CATACGAATATCCCCAGTACTACC | Liu et al.’s [3] | 94°C-3 min, [94°C-30 sec, 52°C-45 sec, 72°C-60 sec]35 loops, 72°C-7 min |
|  | RH1R | GCTTGTTCATGCAGATGTAGATGC | Liu et al.’s [3] |  |
| IRBP | IRBP101Fd | TCMTGGACAAYTACTGCTCACC | Liu et al.’s [3] | 94°C-3 min, [94°C-30 sec, 52°C-45 sec, 72°C-60 sec]35 loops, 72°C-7 min |
|  | IRBP1001Rb | GGAAATGCATAGTTGTCTGCAA | Liu et al.’s [3] |  |
|  | IRBP155F | TKCTCACCAGAAARACTCACRGG | Liu et al.’s [3] |  |
|  | IRBP1021R | AGTTGTCTGCAACMAGTGYSGC | Liu et al.’s [3] |  |
| myh6 | myh6_F507 | GGAGAATCARTCKGTGCTCATCA | Li et al.’s [4] | 94°C-3 min, [94°C-30 sec, 52°C-45 sec, 72°C-60 sec]35 loops, 72°C-7 min |
|  | myh6_R1322 | CTCACCACCATCCAGTTGAACAT | Li et al.’s [4] |  |
| EGR2B | E2B287Fd | TTGACTCSCAGTATCCAGGTAAC | Liu et al.’s [3] | 94°C-4 min, [94°C-40 sec, 53°C-40 sec, 72°C-65 sec]35 loops, 72°C-7 min |
|  | E2B1117Rb | AGGTGGATTTTGGTGTGTCTYTT | Liu et al.’s [3] |  |
| RAG1 | RAG1F1 | AGCTGCAGTCAGTAYCACAAGATGT | Liu et al.’s [3] | 94°C-4 min, [94°C-30 sec, 52°C-40 sec, 72°C-90 sec]35 loops, 72°C-7 min |
|  | RAG1.4090R1 | CTGAATTCTTGTGAGCCTCCATRAAC | Liu et al.’s [3] |  |

**Table S2** GenBank accession numbers for specimens included in this study.

| **Sample ID** | **Species** | **Test number** | **Cyt *b*** | **16S** | **EGR2B** | **IRBP** | **myh6** | **RAG1** | **RH1** |
| --- | --- | --- | --- | --- | --- | --- | --- | --- | --- |
| **Focal taxon** | |  |  |  |  |  |  |  |  |
| 1 | *Triplophysa stoliczkae* | F1398 | MG725405 | MG735526 | MG725429 | MG725471 | MG725509 | MG725547 | MG725589 |
| 2 | *T*. *stoliczkae* | F1910 | MG725407 | MG735531 | MG725434 | MG725477 | MG725515 | NA | MG725595 |
| 3 | *T*. *stoliczkae* | F940 | MG725408 | MG735512 | MG725456 | MG725494 | MG725532 | MG725574 | MG725612 |
| 4 | *T*. *stoliczkae* | F1396 | MG725404 | MG735525 | MG725428 | MG725470 | MG725508 | MG725546 | MG725588 |
| 5 | *T*. *stoliczkae* | F1407 | MG725406 | MG735527 | MG725430 | MG725472 | MG725510 | MG725548 | MG725590 |
| 6 | *T*. *stoliczkae* | F1267 | MG725381 | MG735516 | MG725419 | MG725461 | MG725499 | MG725537 | MG725579 |
| 7 | *T*. *stoliczkae* | F1268 | MG725382 | MG735517 | MG725420 | MG725462 | MG725500 | MG725538 | MG725580 |
| 8 | *T*. *stoliczkae* | F1025 | MG697582 | MG698210 | NA | MG698822 | MG698521 | MG725535 | MG697590 |
| 9 | *T*. *stoliczkae* | F1026 | MG725380 | MG735515 | NA | MG725460 | MG725498 | MG725536 | MG725578 |
| 10 | *T*. *stoliczkae* | F963 | MG725383 | MG735513 | MG725457 | MG725495 | MG725533 | MG725575 | MG725613 |
| 11 | *T*. *stoliczkae* | F964 | MG725384 | MG735514 | MG725458 | MG725496 | MG725534 | MG725576 | MG725614 |
| 12 | *T*. *stoliczkae* | F1388 | MG725400 | MG735524 | MG725427 | MG725469 | MG725507 | MG725545 | MG725587 |
| 13 | *T*. *stoliczkae* | F1305 | MG725398 | MG735520 | MG725423 | MG725465 | MG725503 | MG725541 | MG725583 |
| 14 | *T*. *stoliczkae* | F1283 | MG725394 | MG735518 | MG725421 | MG725463 | MG725501 | MG725539 | MG725581 |
| 15 | *T*. *stoliczkae* | F1285 | MG725395 | MG735519 | MG725422 | MG725464 | MG725502 | MG725540 | MG725582 |
| 16 | *T*. *stoliczkae* | F1351 | MG725399 | MG735521 | MG725424 | MG725466 | MG725504 | MG725542 | MG725584 |
| 17 | *T*. *stoliczkae* | F1365 | MG725396 | MG735522 | MG725425 | MG725467 | MG725505 | MG725543 | MG725585 |
| 18 | *T*. *stoliczkae* | F1367 | MG725397 | MG735523 | MG725426 | MG725468 | MG725506 | MG725544 | MG725586 |
| 19 | *T*. *stoliczkae* | F937 | MG725392 | MG735510 | MG725455 | MG725492 | MG725530 | MG725573 | MG725610 |
| 20 | *T*. *stoliczkae* | F938 | MG725393 | MG735511 | NA | MG725493 | MG725531 | NA | MG725611 |
| 21 | *T*. *stoliczkae* | F931 | MG725390 | MG735508 | MG725453 | MG725490 | MG725528 | MG725571 | MG725608 |
| 22 | *T*. *stoliczkae* | F932 | MG725391 | MG735509 | MG725454 | MG725491 | MG725529 | MG725572 | MG725609 |
| 23 | *T*. *stoliczkae* | F929 | MG725389 | MG735507 | MG725452 | MG725489 | MG725527 | NA | MG725607 |
| 24 | *T*. *stoliczkae* | F928 | MG725388 | MG735506 | MG725451 | MG725488 | MG725526 | MG725570 | MG725606 |
| 25 | *T*. *stoliczkae* | F2564 | MG725409 | MG735534 | MG725440 | MG725480 | MG725518 | MG725558 | MG725598 |
| 26 | *T*. *stoliczkae* | F2565 | MG725410 | MG735535 | MG725441 | MG725481 | MG725519 | MG725559 | MG725599 |
| **Other *Triplophysa* species** | |  |  |  |  |  |  |  |  |
| 27 | *T*. *rotundiventris* | F2077 | MG725402 | MG735533 | MG725437 | MG725479 | MG725517 | MG725555 | MG725597 |
| 28 | *T*. *chondrostoma* | F37 | MG725403 | MG735503 | MG725445 | MG725483 | MG725521 | NA | MG725601 |
| 29 | *T*. *leptosoma* | F1721 | MG725401 | MG735529 | MG725432 | MG725475 | MG725513 | MG725551 | MG725593 |
| 30 | *T. orientalis* | F3134 | MG725414 | MG735536 | MG725442 | MG725482 | MG725520 | MG725560 | MG725600 |
| 31 | *T*. *strauchii* | F735 | KX373853 | MG698223 | MG725448 | MG698836 | MG698532 | MG725567 | MG697906 |
| 32 | *T*. *dorsalis* | F740 | MG725413 | MG735505 | MG725450 | MG725487 | MG725525 | MG725569 | MG725605 |
| 33 | *T*. *tenuis* | F2202 | MG697584 | MG698215 | MG725438 | MG698827 | MG698526 | MG725556 | MG697626 |
| 34 | *T*. *bleekeri* | F3202 | KX373847 | MG698218 | MG725443 | MG698830 | MG698529 | MG725561 | MG697779 |
| 35 | *T*. *brevicauda* | F150 | MG725386 | MG735504 | NA | MG725473 | MG725511 | NA | MG725591 |
| 36 | *T*. *tibetana* | F1540 | MG725387 | MG735528 | NA | MG725474 | MG725512 | MG725549 | MG725592 |
| 37 | *T*. *aliensis* | F1 | MG725412 | MG735502 | MG725418 | MG725459 | MG725497 | NA | MG725577 |
| 38 | *T*. *stenura* | F1638 | MG697583 | MG698212 | MG725431 | MG698824 | MG698523 | MG725550 | MG697592 |
| 39 | *T*. *stewarti* | F2009 | MG725411 | MG735532 | MG725435 | MG725478 | MG725516 | MG725553 | MG725596 |
| 40 | *T*. *microps* | F1775 | MG725385 | MG735530 | MG725433 | MG725476 | MG725514 | MG725552 | MG725594 |
| 41 | *T*. *robusta* | F323 | MG697368 | MG697992 | MG725444 | MG698712 | MG698406 | MG725562 | MG697781 |
| 42 | *T*. *siluroides* | F2458 | MG697459 | MG698085 | MG725439 | MG698571 | MG698259 | MG725557 | MG697629 |
| 43 | *T*. *anterodorsalis* | F3894 | MG725417 | MG735539 | NA | MG725485 | MG725523 | MG725564 | MG725603 |
| 44 | *T*. *markehenensis* | F3893 | MG725416 | MG735538 | NA | MG725484 | MG725522 | MG725563 | MG725602 |
| 45 | *T*. *strauchii* | F736 | KX373854 | MG698224 | MG725449 | MG698837 | MG698533 | MG725568 | MG697907 |
| 46 | *T*. *scleroptera* | F2065 | KX373840 | MG698214 | NA | MG698826 | MG698525 | NA | MG697623 |
| 47 | *T*. *scleroptera* | F2073 | KX373833 | MG698225 | MG725436 | MG698838 | MG698534 | MG725554 | MG697908 |
| 48 | *T*. *orientalis* | F68 | MG725415 | MG735537 | MG725447 | MG725486 | MG725524 | MG725566 | MG725604 |
| 49 | *T*. *rosa* | F3911 | MG697587 | MG698220 | MG725446 | MG698832 | MG698535 | MG725565 | MG697868 |

**Table S3** The optimal partitioning scheme and substitution models for each locus.

| **Locus** | **Loci location (bp)** | **Partitioning scheme** | **Best model** | **Model in BEAST** |
| --- | --- | --- | --- | --- |
| mtDNA | Cyt *b*, 1-1140 | p1 = 1-1140 p2 = 1141-2165 | p1, TrN+I+G p2, HKY+I+G | GTR+I+G |
|  | 16S, 1141-2165 |  |  |  |
| nuDNA | RH1, 1-712 | p1 = 1-712, 1370-2082 p2 = 713-1369, 2083-3587 p3 = 3588-4341 | p1, TrNef+I p2, K80+G p3, F81+I |  |
|  | IRBP, 713-1369 |  |  |  |
|  | myh6, 1370-2082 |  |  |  |
|  | RAG1, 2083-3587 |  |  |  |
|  | EGR2B, 3588-4341 |  |  |  |
| RH1 |  |  | TrNef+I | TrNef+I |
| myh6 |  |  | TrNef+I | TrNef+I |
| IRBP |  |  | K80+G | K80+G |
| RAG1 |  |  | K80+G | K80+G |
| EGR2B |  |  | F81+I | HKY+I |

**References**

1. Xiao W, Zhang Y, Liu H. Molecular systematics of Xenocyprinae (Teleostei: Cyprinidae): taxonomy, biogeography, and coevolution of a special group restricted in East Asia. Mol Phylogenet Evol. 2001;18:163-173.

2. Li J, Wang X, Kong X, Zhao K, He S, Mayden RL. Variation patterns of the mitochondrial 16S rRNA gene with secondary structure constraints and their application to phylogeny of cyprinine fishes (Teleostei: Cypriniformes). Mol Phylogenet Evol. 2008;47:472-487.

3. Liu S, Mayden RL, Zhang J, Yu D, Tang Q, Deng X, Liu H. Phylogenetic relationships of the Cobitoidea (Teleostei: Cypriniformes) inferred from mitochondrial and nuclear genes with analyses of gene evolution. Gene. 2012;508:60-72.

4. Li C, Ortí G, Zhang G, Lu G. A practical approach to phylogenomics: the phylogeny of ray-finned fish (Actinopterygii) as a case study. BMC Evol Biol. 2007;7:44.
